# Answer to Ietros

**Published:** 1805-03-01

**Authors:** 


### *To the Editors of the Medical and Physical Journal.*

GENTLEMEN,

AMONG the publications devoted to the advancement of science or professional practice, there is, perhaps, none in which that end has been so successfully pursued, as the MEDICAL and PHYSICAL JOURNAL. It has been the first to propagate every remarkable invention or discovery lately added to surgery or medicine; it has become a centre of professional correspondence for the whole medical World. Through the medium of this Work, you have deservedly gained an authority that renders your approbation, to a man of scientific or professional ambition, highly desirable—your express or implied censure, what every practitioner in medicine would most anxiously strive to escape

Without moderation, delicacy, and prudence, you could not, Gentlemen, have raised your publication to such eminence; nor have acquired so very extensively the confidence of your brethren in the same profession. It is a task of no common delicacy to please, for so long a period, such a number of correspondents and readers; to watch so effectually against being made the instruments of the interested views, the prejudices, the mutual jealousies, which naturally arise among such an host of professional men; to maintain that elevated propriety of conduct as Editors, which can alone give general satisfaction, without any sacrifice of impartiality, truth, or steadiness, in the prosecution of the proper objects of an undertaking such as yours.

And yet, Gentlemen, you must pardon me the observation, that even your vigilance and candour have not been in every instance exempted from the accustomed fate of all human excellence. Your desire to vindicate the dignity of medical practice; or reluctance, perhaps, to exclude *even unsuitable communications* from a Correspondent, who, for aught I know, may be in other respects highly respectable; has betrayed you to admit into some late Numbers of your Work, a series of LETTERS "*On* QUACKS *and* EMPIRICISM," which I should hope, that few arguments can *now* be necessary to prove to you, to have been MUCH TOO INCONSIDERATELY HAZARDED. Be not offended with the frankness of this remark: it is not offered to insult either your judgment or your honour. *Aliquando*

*bonus*

*bonus dormitat Homerus.* You stand greatly above the want of my *praise:* and you are much too liberal in your sentiments, not to forgive a little friendly censure hinted to you with deference and respect.

In his fifth letter, your Correspondent pretends to give the history of Dr. WILLIAM BRODUM. In honour, in candour, you will not deny me the indulgence of calling your attention to the injustice and inconsistency of what he has made the MEDICAL and PHYSICAL JOURNAL, a channel to communicate respecting this well-known practitioner in Medicine.

If I mistake not your Correspondent's aim, he wishes to brand Dr. BRODUM as a QUACK and EMPIRIC, by representing his BIRTH, his EDUCATION, his RELIGION, his FAVOURITE MEDICINES and PROFESSIONAL PRACTICE; but especially the arts of advertisements and popularity, by which he has risen to wealth and reputation, as all conspiring to render it impossible that he should prescribe with success or safety.

I. The FIRST thing which astonishes me, in considering this attack on a living practitioner in medicine, is, that so many opprobrious assertions should be offered, without the declaration of the name of him who makes them; without exact reference to the authority of any other credible witness; in fact, without one grain of evidence, but what is to be found in the *ipse dixit* of an anonymous writer, who is plainly too warm with passion, too frequent in self-contradictions, too careless in his references even to printed authorities, to leave it possible for candour to receive, upon his testimony, one proposition, however trivial, that he does not demonstrate by incontrovertible extrinsic proofs.

II. Dr. BRODUM'S BIRTH and RELIGION are among the first subjects on which turns your Correspondent's reproach. "The doctor was the son of Jewish parents, was born on the Continent, has become a convert to the Christian faith."—He *was* born a Jew! and is it so dishonourable to be of the same national descent, which was preferred by the Son of God, when he revealed himself on earth for the salvation of mankind? Is it not to the *immediate communication* of men, who were all by nation Jews, that we owe the Holy Scriptures, the fundamental code of all divine and human wisdom? Who but Jews were the most eminent physicians, chemists, surgeons, and apothecaries, in the earliest ages of the practice of these several professions in modern Europe? Even the nobility of England and Ireland, the highest in the world, derive honour from

 the the families of Jewish descent and alliance which might be named among them. Among those eminent gentlemen, bankers, and merchants, whose presence constitutes the pride and prosperity of the city of London, how very considerable a proportion are Jews? Even your Correspondent himself has not failed to mention with due honour, the names of a Goldsmid, a Solomon, a Sequeira, a Myers, an Eliaçon! Surely, gentlemen, when the prejudices of rank, religion, national diversity, trade and profession, have been generally exploded, which, to the common mischief of humanity, so long divided man from man, and inflamed the heart of one son of Adam against another; it is not for you, or your friends, to maintain, that "*Dr.* BRODUM *cannot be a skilful and liberal physician, merely because he was born a* JEW.

But, still worse! "*Dr.* BRODUM *was born in Copenhagen!*" The assertion is given on no authority; and it is utterly false! Yet, what, if he had been born in Copenhagen? Is this also a reproach? Is this another of your Correspondent's reasons, why it is impossible that Dr. BRODUM can be a skilful physician? And was it impossible for this Novelist of Medical Biography to wreak his spleen without insulting the natives of a country, to which so large a proportion of the old gentry and yeomanry of England trace back their ancestors? A country that, of any other in Europe, has certainly furnished its full complement of men, eminent not only in medicine, but in every branch of erudition, science, and arts!

Above all, your Correspondent is indignant against Dr. Brodum, *because he has become a convert to the Christian faith.* What! because the Doctor was born of Jewish parents, he is not to be allowed to practise medicine reputably! Because, like Luke, the evangelist and physician, he has examined and embraced Christianity, he cannot be a good physician! What would this calumniator have? "Brodum mustbe neither a Jew nor a Christian." Yes, it is too plainly against the success, against the very existence of Dr. Brodum, that this fierce and inconsistent *bellum ad internecionem* is waged. He must not be either Jew or Christian, because he has been fortunate in the practice of medicine, and in the sale of some popular remedies!

"But he obtained the degree of Doctor in Medicine by DISINGENUOUS PRETENCES; and obtained it from the University of Aberdeen; an University that deals out its medical honours without having due regard to the talents and merits of the candidates."

Both

Both these assertions, Gentlemen, appear from the very tenor of your Correspondent's own narrative, not to be true. Your Correspondent himself says, that Dr. Brodum's "*genius and perseverance surmounted every obstacle*" that stood in the way of his promotion to the highest medical honours. But, it is essential to the very existence of TRUE GENIUS to be incapable of descending to little dishonest arts. He allows, that Dr. Leo recommended Dr. Brodum to those attentions of science, friendship, and professional honour, to which he owed his degree as Doctor in Medicine. Dr. Leo's discernment or integrity he has not dared to impeach; and we have no reason to think that he would have spared a gentleman who was so much the friend of Dr. Brodum, had not that gentleman's character and abilities been altogether blameless and unassailable. But, if Dr. Leo was neither so weak-minded as to be himself imposed upon; nor so false and deceitful, as knowingly to impose upon others: it must of necessity follow, that there was no imposture, no disingenuous pretence, no art of which a man of honour can have reason to be ashamed, in the steps which were taken to procure for Dr. Brodum those medical honours which your Correspondent envies him. But it was not Dr. Leo alone who must either *have imposed*, or *have been imposed upon*, if Dr. Brodum was undeservedly recommended to the University of Aberdeen. Brodum's talents, his knowledge, his professional experience, his authenticated certificates of character, were examined by *another* physician, one of the best and most discerning men, and one of the most eminent physicians in this or any country,—examined by him,—and approved.

Yet a *third time*, his claims to the honour of graduation were to be examined, and by a body of the most upright, ingenuous, and judicious men, of whom science and learning have ever, in any country, had to boast,—by the members of the University of Aberdeen. Thus Brodum's claims had to pass, successively, through three different ordeals. Thrice were they investigated by persons, not in conspiracy to support them, not interested to allow them at all hazards,—notoriously incapable of being easily deceived themselves, and infinitely above the suspicion of becoming parties to any deceit that should impose upon others. How dares your Correspondent to throw out, in such a case, the aspersion of imposture? Knows he any thing at all of the laws of Evidence, or the possibilities of human conduct? Surely, if he does; he he must be ashamed to have ever advanced any thing insinuating, that there was *imposture* in the manner in which Dr. Brodum obtained his degree.

I cannot pass from this part of my observations without observing in Dr. Brodum's favour, that, by your Correspondent's own acknowledgment, his right to the honour of Doctor in Medicine, came, even a fourth time, under investigation, when he began to recommend his famous patent medicine by frequent advertisements in the newspapers. The delicacy of some of the members of the University of Aberdeen took a momentary offence at this; they were induced anew, to inquire whether they had not been imposed upon when they honoured the Doctor with his degree. There was a disposition to find out something against the Doctor. The University inquired of themselves; they wrote to request that the physicians who had recommended Brodum to their notice, would renew their inquiries. All this was done; and the result only satisfied the University, that they had done well at first; and that Brodum could do them nothing but honour in wearing the dignity with which they had invested him. How can your Correspondent be so absurdly outrageous, as to question Brodum's title to honours which he has not obtained and preserved without as regular and as impartial an examination of his right to them, as ever any medical man was subjected to?

III. The next charge which this writer has introduced into the Medical and Physical Journal, relates to the medicines which Dr. Brodum sold; to the arts by which he raised himself to notoriety and fame; to the destruction of lives, which the calumniator would insinuate that the doctor occasioned by his practice, after he had been so honoured by the University of Aberdeen. The Doctor's "*Nervous Cordial,*" and his "*Botanical Syrup,*" are described as useless nostrums; powerless to do good or harm; preparations from vegetable substances, long before known in medicine, and included among the formulary prescriptions of physicians. Yet, in a rhapsody about Judas Iscariot, and the Witch of Endor, and the Professors of Aberdeen, it is more than insinuated that Dr. Brodum has destroyed his patients by them. And that same Correspondent of yours, who says, that the *Botanical Syrup* is a preparation long famous among regular physicians, scruples not elsewhere to allege, that Dr. Brodum was guilty of fraudulent quackery in recommending it so boldly by public advertisement.

To

To these daring accusations, Gentlemen, I would, by your leave, answer,

1. That Dr. Brodum's "Nervous Cordial," and "Botanical Syrup," are most certainly nothing more than particular mixtures and combinations of ingredients of vegetable origin, which, both separately and in composition, have been, for centuries, among the most famous remedies in which the power of medicine has been confessed. They are invigorating and nourishing. They have been known, ever to act with the most salutary efficacy upon constitutions in a state of general relaxation and decay. They have never gone into discredit. The more that they have been analysed by chemistry; the more that they have been tried under different theories of medical opinion; so much the more has their fame increased. At present, when bark, wine, and so many saccharine and mucilaginous preparations are so much in use, these very calumniated remedies of Brodum's are more than ever allied to the best and most fashionable medical practice.

2. The "Nervous Cordial," and "Botanical Syrup," were never prescribed by Dr. Brodum, mysteriously or with a secrecy, as if he had been afraid of being detected in something wrong if it should have come to be known how he prepared them. He obtained his *Patent Right*, not till after the Law-Officers of the Crown had duly enquired into the innocence and usefulness of the preparations of which the property was so secured to him. His *specification* was duly enrolled; and left, for the satisfaction of the public, at the proper offices. If the preparations were not of Brodum's invention? why does no other person come forward, quote the specification, and demonstrate that it specifies nothing new? If those preparations were noxious; why did no humane and patriotic physician; why did not this zealous and passionate Correspondent of yours, stand forth, quote the specification, and shew, that the author deserved punishment as a poisoner; not honor and reward as an inventor,—one of those great men, *qui vitam excoluere per artes?*

3dly. If any have ever perished victims to the Doctor's inexperience, or to the pernicious quality of his medicines; why have there been no complaints? Were there none who left surviving friends or relations to resent their death, and warn others against confiding health and life to the man by whose guilt and ignorance those persons had fallen? How is it that the reputation of Dr. Brodum himself, and of his remedies, has gone on from year to year increasing? How is it that your Correspondent has not one unfortunate case out of the whole period of Dr. Brodum's practice, to produce against him?

4thly. The same things cannot be both noxious and uselessly harmless. In ascribing such contradictory qualities to Brodum's medicines, your Correspondent evinces that he is not more eager to accuse than destitute of all matter of accusation.

5thly. Dr. Brodum wrote prescriptions, and gave directions for care and regimen, at the same time that he sold his Patent-Medicines. He did not trust to the blind use of his remedies, without any distinction of cases and of circumstances. Let his prescriptions and directions be produced against him, if they were those merely of poisoning, assassinating quackery! Have none of them been preserved? Has the keenness of your Correspondent's malice been able to discover nothing of this sort that could have justified his accusations? Or, how can it be consistent with reason and justice, to blame that man as a quack, who gave advice and prescriptions together with his medicines; and whose advice and prescriptions no one can, by *specific* and direct facts, prove to have been ignorant or injudicious?

6thly. To seek professional notoriety by advertisements in the newspapers, is said by many of those who value themselves on the character of regular physicians, to be wrong. But, what are the occasions so industriously sought for nominal mention in the newspapers, the whispers against a neighbour's fame, and to one's own honour? what are these, and the innumerable other arts or means of artifice, in common use among active young physicians,—but so many less open, less ingenuously frank, and by consequence less honourable, and more discreditable Advertisements? The use of newspaper advertisements between those who want aid or accommodations, and those who can supply them, is not a shift of imposture, but a grand improvement in the facilities of trade and social intercourse. No knave can appeal to the public in advertisements with impunity. Societies, œconomical, scientific, and charitable; merchants throughout all the departments of trade, wholesale and retail; the government of every free country, in all its transactions of domestic management or foreign arrangement; men in every condition of life, and in every mode of professional employment; have considered it as perfectly honourable and convenient to seek seek that public notoriety through the channel of the news papers.

We sometimes see at the Old Bailey, that a witness labouring to prevaricate and swear falsely, betrays himself unawares, by revealing some truth which destroys his whole system of perjuries; and such has been the fate of your Correspondent! He allows to Brodum, genius, shrewd sagacity, a love of fame, a taste for magnificence, a heart open to charity! And these are qualities which the Doctor undeniably possesses. But is it possible, that these qualities should co-exist in the same character with the foolish, the odious, the criminal ones, which your Correspondent, at the same time, imputes to him? Of such an union, there is certainly no recorded example, even from Adam down to Dr. Brodum. A few grains of common sense might have taught your Correspondent to preserve a more plausible congruity in his fictions. But this imperfection is just such another as that by which he runs into such unreasonable digressions; quotes books which never existed; ascribes the composition of Gil Blas to the Marquis D'Argens; and finds the geographical situation of a fountain of Arcadia, in America!

Dr. Brodum's late visit to those parts of the Continent, where he had lived in early life, affords still another topic of falsehood and reproach to your Correspondent. Nor can he display his malice here against Brodum, without going to disturb the ashes of the Empress-Queen of Germany, and of the late King of Prussia. This accuser blames the Doctor as having been guilty of an heinous crime in taking letters of credit to secure to him ample supplies of money, while he should be on his travels! Ridiculous! He would, no doubt, have been much better pleased, if Brodum had been obliged to go abroad to starve! But what was there contemptible? what was there dishonest? what was there ridiculous? in his taking in his pocket a letter of credit for fifty thousand rix-dollars? Was Dr. Brodum to blame, if Dr. F's letter of credit was for 100l. only? Dr. Brodum's tour was one of pleasure and of liberal and scientific enquiry, not at all one of ambition. Whereever he went, he met from the great and the learned, that flattering reception to which his manners, his figure, and his celebrity entitled him. His notice was courted by men of science in Russia. He lived on friendly terms with the most eminent physicians, and with noblemen of the first fashion, at Copenhagen. At Berlin, his conversation was acceptable in the highest circles. At Paris,

Paris, Bonaparte readily gave him audience; Fourcroy honoured his medicines with a sanction which has never been prostituted to any unworthy purpose; Chaptal gave him a flattering reception; and he found Berthollet partial to his, as to every other invention that came from England.

Allow me, now, Gentlemen, to conclude with mentioning a few *real* particulars of Dr. Brodum's life. He was born and received his early education near Heidelberg, in Germany. He was instructed in medicine and surgery at several Universities in the Dutch Provinces. He then served as a surgeon's mate and as a surgeon in the navy of Holland during the American war. He was present in the engagement off the Dogger-Bank. He went in the same service to the West Indies. In Surinam, he gained reputation and money by remarkable success in the cure of dysentery. He came afterwards, by the chance of service, into employment among his countrymen, the Hessian troops, who were in the British service in America. On his return to Europe, he went for a time to the Continent, there spent the savings of his service abroad, and then returned almost pennyless to England, and with little or no knowledge of the English language. In these unhappy circumstances, he was glad to become assistant to Mr. Lamert. After a few months, he left Mr. Lamert, and went to try his fortune, alone, at Portsmouth. Lamert recalled him to London, with offers of equal partnership. He soon aspired above the practice and reputation of his partner; obtained suitable recommendation from his masters in Holland; and upon those, and other satisfactory testimonies, the honour of graduation by the University of Aberdeen. He then procured the king's letters-patent, to secure to him the sole property of his *nervous cordial* and *vegetable syrup;* and courted fame and business by frequent advertisements in the news-papers. His medicines became known: the prescriptions with which he accompanied them were valued still more highly. Fame, fortune, and envious abuse have followed. Dr. Brodum having risen to opulence, amid splendid expence, has some time since sold his patent medicines, and his whole business as a medicine vender. Some time after he found himself rich and free, was given to a journey to those scenes which he delighted to remember on the Continent. But, he could not think to settle for the remainder of his life elsewhere than in England. He now resides in a very handsome house in Bernard Street, Brunswick-square, Bloomsbury. He lives in the social converse of his his friends, among whom he numbers most men of professional or fashionable eminence in London; and in such practice of his profession, as his former celebrity still continues to force upon him, even while he would gladly shun it. The weakest, but the most malicious attack ever made upon him was in January last by your correspondent, whom I have taken the trouble to refute. Dr. Brodum only laughed, and cried, "Go! go! poor devil! sure there is room enough for thee and me both in the wide world." But his friends have insisted, that it is for the interest of truth and of charity, that calumnies so gross should be expressly refuted. It is for this purpose I address myself to your honour and candour, Gentlemen, and earnestly wish your immediate insertion of what I have here written. In this, you will exceedingly oblige,

Your's, &c.

*****

---

P. S. I have subscribed my name for your satisfaction, and that you may communicate it upon suitable private inquiry; but I should not wish it to be given to the public till the calumniator, whose abuse it refutes, shall openly come forward.

---

The following is a copy of a letter from Dr. Leo to the Editors. The original is in the hands of the publisher.

"Gentlemen,

"Reading in the Letter 'Of Quacks and Empiricism,' in No. 71, of the Medical and Physical Journal, signed Ietros, that *Dr. Leo was made the tool to carry on Dr. Brodum's impostures;* you will, I trust, do me the justice to contradict such assertion, when I assure you, that my exertion in favor of Dr. Brodum, arose from various letters of medical gentlemen on the Continent, whom I knew, particularly from Dr. D'Haen of Utrecht, Dr. Nierop of Amsterdam, and Dr Meyers of Surinam, at which latter place the Doctor had practised with success, strongly recommending the Doctor's professional skill and abilities to my notice, whose interest as a foreigner and a stranger, with such recommendations from my friends, I considered as no more than my duty to espouse without fee or reward.

"I am, &c.

"L. LEO, M.D.

"Physician to the Portuguese Hospital, &c. &c."

CRITICAL

